# The gut microbiota heterogeneity and assembly changes associated with the IBD

**DOI:** 10.1038/s41598-018-37143-z

**Published:** 2019-01-24

**Authors:** Yang Sun, Lianwei Li, Yao Xia, Wendy Li, Kunhua Wang, Lan Wang, Yinglei Miao, Sam Ma

**Affiliations:** 1 Department of Gastroenterology, The First Affiliated Hospital of Kunming Medical University, Yunnan Institute of Digestive Disease, Kunming, Yunnan Province China; 2 Computational Biology and Medical Lab, State Key Laboratory of Genetic Resources and Evolution, Kunming Institute of Zoology, Chinese Academy of Sciences, Kunming, China; 3 Department of General Surgery, The First Affiliated Hospital of Kunming Medical University, Yunnan Institute of Digestive Disease, Kunming, Yunnan Province China

## Abstract

Inflammatory bowel disease (IBD) is an immunologically mediated disease and may be caused by abnormal immunological response to gut microbes. Although several studies on the ecological changes associated with IBD, such as community diversities, were reported, no previous studies have investigated the changes in the spatial heterogeneity and the mechanism of community assembly of the gut microbiota associated with IBD. In the present study, we first applied the Taylor’s power law extensions to compare the community spatial heterogeneity between the gut microbial communities of the IBD patients and those of the healthy individuals. We found that the community spatial heterogeneity of gut microbiota in IBD patients is slightly lower than in the healthy individuals. This finding suggests that IBD may lower the spatial heterogeneity of gut microbiota, possibly *via* lowering the abundance of dominant species. We further applied the neutral theory of biodiversity to comparatively investigate the community assembly and diversity maintenance of the gut microbiota with and without IBD, and our application suggested that deterministic factors such as host immunity should be dominant forces shaping gut microbiota assembly, and diseases such as IBD may not be strong enough to change the trend set by the deterministic host factors.

## Introduction

Inflammatory bowel disease (IBD) is a series of inflammations of colon and small intestine, consisting of two principal types, Crohn’s disease (CD) and ulcerative colitis (UC)^1,2^. Besides small intestine and large intestine, CD can also affect other parts of human body, such as stomach and anus, whereas UC is generally limited to colon and rectum^3–5^. Although the cause of IBD remains limitedly understood, the most widely accepted hypothesis is that IBD is caused by abnormal immunological response to gut microbes, where the environmental factors and the genetic susceptibility of host may also play an important role^1,2,6–9^. Obvious differences in the composition of gut microbiota between healthy individuals and IBD patients, and between inflamed and non-inflamed regions of the intestine have been widely reported^8–17^, and such specific changes of composition of gut microbiota have been conceived for developing a promising strategy for the diagnoses of IBD^10,18^. The pathological changes of gut microbiota is usually referred to as dysbiosis and, from the view of ecology, it could be regarded as the instability of microbiota.

The instability (*i.e*. dysbiosis) of microbiota is also well recognized as a sign of IBD progression^19–22^. One way to evaluate the instability is to measure the variations of gut microbiota. High levels of both inter-individual variations within humans and longitudinal variations across time have been reported in several population-based studies^23–25^. From a perspective of inter-individual variations, instability of microbiota is obviously equivalent to its heterogeneity. Although the instability is inevitable, our particular concern is that whether or not IBD could raise (or lower) the degree of heterogeneity (variation) of the gut microbiota. Taylor’s power law (1961)^26^ describing the scaling relationship (*i.e., V* = *am*^*b*^) between the mean (*m*) and variance (*V*) of population abundance offers an effective tool to investigate the population heterogeneity. Ma (2015)^27^ extended Taylor’s power law to community level, which makes it possible to evaluate the community spatial heterogeneity and temporal stability with four power law extensions (PLEs). The PLEs have been applied successfully in several studies. For examples, Zhang *et al*. reported significant spatial heterogeneities existing in the human gut mucosa microbiota using PLEs^28^; Oh *et al*. applied the PLEs to evaluate the temporal heterogeneity of skin microbiota^29^. Our first objective in this study is to apply the PLEs to evaluate the influence of IBD on the community spatial heterogeneity of human gut microbiota.

Another important question of both theoretical and practical significance in community ecology is that whether or not the mechanism of community assembly and diversity maintenance of gut microbiota would be changed due to IBD. In theoretical ecology, the traditional niche theory stipulated that community assembly is governed by deterministic factors, such as competition and niche differentiations^30,31^. An alternative to the deterministic niche theory is the neutral theory^32^ that empathizes stochastic factors. The neutral theory developed by Hubbell^32^, integrates neutrality, stochasticity, sampling and dispersal and formulates a null model to test the mechanism of community assembly and diversity maintenance^33,34^. The neutral theory has been extensively tested in macro-ecology field in the last two decades, but its applicatoions in microbial ecology are relatively new^34–38^. In a previous study, Li & Ma^34^ conducted a comprehensive testing of the neutral theory with the human microbiota project (HMP) datasets that included samples from 18 body sites of 242 individuals. In this article, we apply the Hubbell’s (2001) neutral theory to detect the possible changes in the mechanism of community assembly and diversity maintenance associated with IBD.

Overall, our study focuses on revealing the possible ecological changes in the human gut microbiota associated with IBD. Specifically, we try to investigate the following two important questions that have not been addressed previously to the best of our knowledge: (*i*) whether or not IBD would raise (or lower) the spatial heterogeneity of gut microbiota; (*ii*) whether or not IBD would change the mechanism of gut microbiota assembly and diversity maintenance. To answer the first question, we applied Ma’s (2015)^26^ power law extensions (Type-I and Type-III PLEs) for assessing community (Type-I) and mixed-species (Type-III) spatial heterogeneities. To address the second question, we apply Hubbell’s (2001)^32^ neutral theory of biodiversity and Etienne’s^39^ sampling distribution model for implementing the neutral theory test. Both the analyses for addressing the two questions are performed with the datasets from a comprehensive metagenomic study originally conducted by Papa *et al*.^40^.

## Materials and Methods

### Dataset description

The dataset we reanalyzed is from a comprehensive metagenomic study originally performed by Papa *et al*.^40^, in which they recruited 91 children and young adults and collected their fecal samples for 16S rRNA sequencing, among whom 23 had Crohn’s disease (CD), 43 had ulcerative colitis (UC), one had undefined IBD (colitis with elements of CD and UC) and 24 had non-IBD functional disease (patients with gastrointestinal symptoms but no intestinal inflammation). We also downloaded the raw sequence data from NCBI BioProject, with BioProjectID 82109, and selected a subset with definite status of the healthy, CD^17^, and UC^38^, respectively. We obtained the OTU (Operational Taxonomic Unit) tables by processing the raw data with MOTHUR^41^ according the steps described in Papa *et al*.^40^. In brief, sequences with lengths less than 200 nt or greater than 600 nt were removed, and low quality sequences with quality score less than 25 were also removed. Then sequences contained ambiguous characters, had a non-exact barcode match, or had more than 4 mismatches to the reverse primer reads (926R) were eliminated as well. Remaining sequences were assigned based on barcode matches. Then barcode and primer sequences were trimmed. ChimeraSlayer was adopted to identify Chimeric sequences and MSU RDP classifier (v2.2) was used to classify reads on the basis of the taxonomy maintained at the Ribosomal Database Project (RDP 10 database, version 6). After processing, the resulting sequencing depth was 2571 (mean) reads per sample. And finally the OTUs were clustered at 97% similarity level. Although in 16S rRNA sequencing, an OTU is not limited to referring to a species (e.g. OTU can also be genus or family). In order to keep consistence when referring the original methodological references in ecology field, in the later text, we will use the term “species” to refer to “OTU”. The OTU table, which contains the abundances of each OTU in each community sample, is equivalent to the species abundance distribution data in macro-ecology, and is utilized to assess community spatial heterogeneity and fit the neutral theory model.

### The power law extensions (PLEs)

Taylor’s (1961) discovered the power law relationship is in the following form:

1 $$V=a{m}^{b},$$

where *m* is the mean population abundance and *V* is the corresponding variance, *b* is a species-specific parameter measuring the population aggregation degree, and *a* is a parameter mainly influenced by sampling scheme and is of relatively little biological significance.

Ma (2015)^26^ extended the original Taylor’s^27^ power law to the community level by introducing four power law extensions (PLEs), of which Type-I PLE was extended to measure the *community spatial heterogeneity*. Type-I PLE possesses the same mathematical formula with the original Taylor’s power law, but with different ecological interpretations for both the variables and parameters, *i.e*.,

2 $${V}_{s}=a{{m}_{s}}^{b}\,s=1,\,2,\,\ldots ,\,S$$

where *m*_s_ is the mean of abundances of all species at the *s-th* sampling site, *i.e*., the mean species (population) size (abundance) per species, *V*_s_ is the corresponding variance, *S* is the number of total sampling sites, *a* is similarly interpreted as in the original Taylor’s power law, and parameter *b* measures the community spatial heterogeneity.

Type-III PLE was proposed to measure the spatial heterogeneity of the mixed species population. It also possess the same mathematical function with the original Taylor’s power law and Type-I PLE, but with different ecological interpretations for the model variables and parameters, *i.e*.,

3 $${V}_{m}=a{{m}_{m}}^{b}\,m=1,\,2,\,\ldots ,\,{M}$$

where *m*_*m*_ is the mean of population abundances of a specific species across all samples (sites), *V*_*m*_ is the corresponding variance, *M* is the number of species in the community, *a* is related to the sampling scheme and of little biological implications, and *b* measures the spatial heterogeneity of mixed-species.

Both Type-I and Type-II PLEs can be fitted to datasets in the same manner as the original Taylor’s power law was fitted (Taylor 1961)^27^ *i.e*., using the following log-transformation:

4 $$\mathrm{ln}(V)=\,\mathrm{ln}(a)+b\,\mathrm{ln}(m)$$

Ma (2015)^26^ further proposed the concept of community heterogeneity critical diversity (CHCD) and derived its formula as

5 $$CHCD=\exp (\frac{\mathrm{ln}(a)}{1-b}) > 0,\,b\ne 1)$$

where *a* and *b* are the parameters of the PLEs. CHCD is a threshold or transition point between heterogeneous community and regular (completely even) community, and at the point of CHCD, the community is random. As a side note, Type-II and IV PLEs are related to temporal heterogeneity and are not implicated with this study.

### Hubbell’s (2001) neutral theory of biodiversity

We used the sampling formula proposed by Etienne^39^, which adopts a maximum likelihood method and enhances the classic Ewens sampling formula^42^ by adding a dispersal limitation, to estimate the key parameters of the neutral theory model, *i.e*., the fundamental biodiversity parameters *θ* and migration probability *m* (*m* < 1). Parameter *m* is defined as:

6 $$m=\frac{I}{I+j-1}$$

where *I* is the number of migrants, *j* is the *j-*th individual of community. For the species abundance distribution *D*, the Etienne sampling formula is in the following form:

7 $$P(D|\theta ,\,m,\,J)=\frac{J!}{{\prod }_{i=1}^{S}{n}_{i}{\prod }_{j=1}^{J}{\varphi }_{j}!}\frac{{\theta }^{S}}{{(I)}_{J}}\sum _{A=S}^{J}K(D,\,A)\frac{{I}^{A}}{{(\theta )}_{A}}$$

where *J* is the total number of individuals in the community, *S* is the total number of species, *θ* is the fundamental biodiversity parameter, *n*_*i*_ is the abundance of species *i*, and *ϕ*_*j*_ is the number of species with certain abundance. *K (D, A)* is defined as:

8 $$K(D,\,A)=\sum _{\{{\alpha }_{1},{\alpha }_{2}\mathrm{...}{\alpha }_{S}|\sum _{i=1}^{S}{\alpha }_{i}=A\}}\prod _{i=1}^{S}\frac{\overline{{S}_{({n}_{i},{\alpha }_{i})}}\overline{{S}_{({\alpha }_{i},1)}}}{\overline{{S}_{({n}_{i},1)}}}$$

We then generated predicted (simulated) community with parameters *θ* and *m* by following the steps below:

(*i*) Generate a vector with length *J*,

9 $$j=1,\,2\,,\,3,\,\mathrm{...}\,J$$

(*ii*) Computing *I*_*m*_,

10 $${I}_{m}=\frac{m(J-1)}{1-m}$$

(*iii*) For each *j* form step (*i*), computing

11a $${U}_{1}={I}_{m}/({I}_{m}+j-1)$$

11b $${U}_{2}=\theta /(\theta +a-1)$$

*U*_1_ and *U*_2_ are used to determine that whether a new individual belong to an existing species or to a new species. We find *x* (0 < *x* < 1) from a uniform distribution, if *x* > *U*_1_, the new individual *j* belong to an existing species. Otherwise, we should get another value *y* (0 < *y* < 1) also from a uniform distribution. Comparing *y* and U_2_, if *y* ≤ *U*_2_, the *j-*th individual is a new species, if *y* > *U*_2,_ this individual also belong to an exist species.

We used the following log-likelihood ratio test to compare the observed community and neutral predicted community:

12 $$D=-\,2\,\mathrm{ln}(\frac{{L}_{0}}{{L}_{1}})=-\,2[\,\mathrm{ln}({L}_{0})-\,\mathrm{ln}({L}_{1})]\,\,\,$$

where, *L*_0_ is the log-likelihood of the null model and *L*_1_ is log-likelihood of the alternative model, *D* is the deviation that is twice the difference between the log-likelihoods of observed and predicted community.

We utilized Etienne’s^43^ exact neutrality test method to test the neutrality of community samples, which can be summarized as the following two steps: (*i*) Use the maximum likelihood estimation (MLE) to estimate the model parameters in terms of the observed samples. For each sample, we simulated 100 artificial communities using parameters (*θ, I, J*) estimated based on the observed samples, and then used Etienne formula to calculate the likelihood for each artificial community, namely *P*_*s*_. (*ii*) Compare the mean value of the likelihood (*P*_*s*_) of 100 artificial communities for each sample and the likelihood (*P*_0_) of corresponding observed sample, by using a chi-squared test under the null hypothesis that there is no significant difference between the probability from observed community and the values computed from the artificial data sets. If no significant difference between *Ps* and *P*0 is detected (*p*-value greater than 0.05), the community would be considered neutral.

## Results and Discussion

### Assessing the change of community spatial heterogeneity associated with IBD

**Figure 1:**
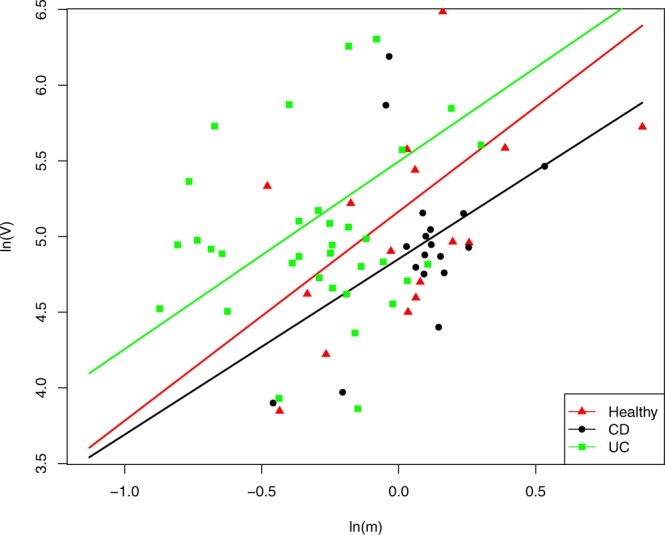
Fitting to the Type-I PLE for the healthy, CD and UC treatments, respectively: the CD & UC treatments showed higher scaling parameter *b*. The *X*-axis represents the logarithm transform of *m*_*s*_, the mean species population size (abundance) *pe*r species in the community at a specific sampling site, and the *Y*-axis represents the logarithm transform of *V*_*s*_, the corresponding variance. The trend lines of scatter plots for *the* healthy, CD and UC treatments are in red with triangles, black with circles and green with squares respectively.

**Figure 2:**
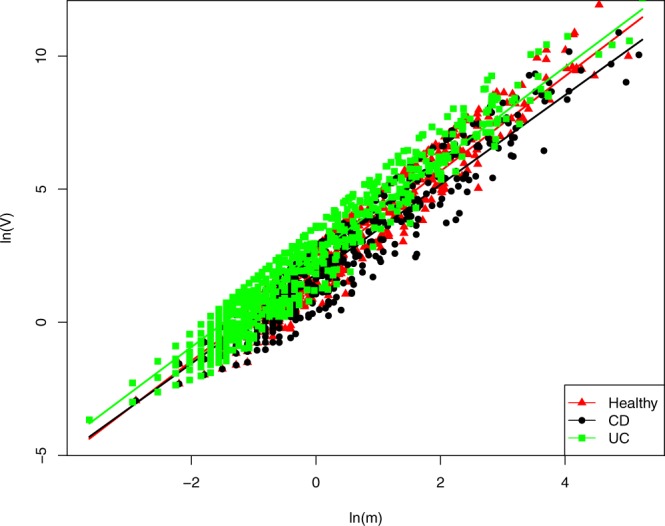
Fitting to the Type-III PLE for the healthy, CD and UC treatments, respectively: no significant differences between the three treatments. The *X*-axis represents the logarithm transform of *m*_*m*_, the mean of population abundances of a specific bacterial species across a series of spatial sites (per site), and the Y-axis represents the logarithm transform of *V*_*m*_, the corresponding variance. The trend lines of scatter plots for the healthy, CD and UC treatments are in red with triangles, black with circles and green with squares respectively.

Table 1 listed the parameters of Type-I and Type-III PLEs fitted to the three treatments (*i.e*., healthy, CD and UC), respectively. In the case of Type-I PLE, the parameter *b* for the healthy treatment is slightly larger than that of the other two treatments (Fig. 1). However, the differences among three treatments in terms of Type-III PLE seemed insignificant (Fig. 2). The Type-I PLE model suggests that IBD may have certain influence on the community spatial heterogeneity, but not on the spatial heterogeneity of mixed species populations. In other words, IBD may influence the community-level spatial heterogeneity, but not the mixed-species level heterogeneity. The former represents the inter-species abundance variations (heterogeneities) exhibited at the community level. The latter represents the population abundance variations (heterogeneities) among spatial sites in terms of the mixed species, which is essentially a species (population) entity or exhibited at the population level.

**Table 1:** Test results of fitting to Type-I and Type-III PLE for the healthy, CD and UC treatments.

PLE	Treatments	*b*	*SE*(*b*)	*ln*(*a*)	*SE* [*ln*(*a*)]	*CHCD*	*R*	*p*-value	*n*
Type-I PLE	Healthy	1.382	0.542	5.165	0.181	0.000	0.537	0.021	18
	CD	1.161	0.626	4.852	0.132	0.000	0.420	0.082	18
	UC	1.240	0.419	5.496	0.181	0.000	0.442	0.005	38
Type-III PLE	Healthy	1.784	0.006	2.110	0.015	0.068	0.985	0.000	2440
	CD	1.680	0.007	1.809	0.016	0.070	0.979	0.000	2424
	UC	1.754	0.006	2.573	0.016	0.033	0.984	0.000	3201

### Assessing the change of community neutrality associated with IBD

**Figure 3:**
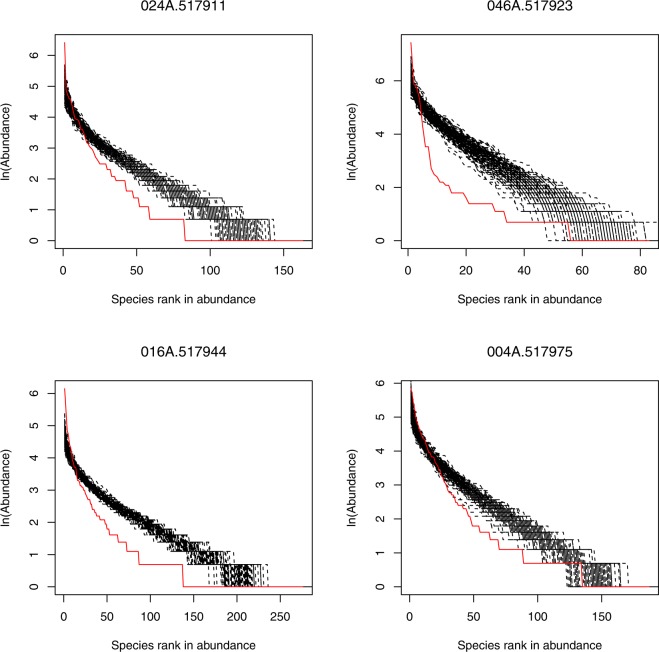
The graphs of four community samples fitted to the neutral theory model of biodiversity. The graphs show poor fitting of the neutral theory model, *i.e*., the significant difference between the actual community observations (red line) with the artificially simulated communities (black lines) based on the neutral theory model.

For each sample we calculated the fundamental biodiversity parameter *θ*, the immigration rate *m* and the corresponding *likelihood* using Etienne formula. Detailed results were listed in the Supplementary Table S1. We compared the likelihood (*P*_0_) of observed dataset and the average likelihood (*Ps*) of corresponding 100 artificial datasets for each sample *via* Etienne formula. About 4.1% (3/74) samples in total satisfied the neutral prediction, and there were around 5.6% (1/18) samples in the healthy, 0% (0/18) in CD and 5.3%(2/38) in UC treatment satisfied the neutral prediction. No significant differences among the three treatments were observed. The parameters for all samples passing the neutrality test were listed in Table 2. Figure 3 showed the graphs of four samples fitting to the neutral theory model. In addition, we also tested 12 healthy communities that were first reported in Halfvarson *et al*.^44^, and all communities failed to pass the neutral test. The detail information of supplementary results was listed in Table S1.

**Table 2:** The parameters of the three samples that passed the neutrality test.

Treatment	ID	J	S	θ	m	p-value
Healthy	005A.517956	2866	101	20.304	0.99980	0.165
UC	120D.517914	1614	65	13.462	0.99986	0.060
	178A.517971	3671	127	25.394	0.99998	0.081

*J*: the total number of reads in the sample, *S*: the number of species in the sample, *θ*: fundamental biodiversity number, *m*: immigration probability, and *p*-value is calculated *via* a chi-squared test for comparing *P*_*s*_ and *P*_*0*_.

**Figure 4:**
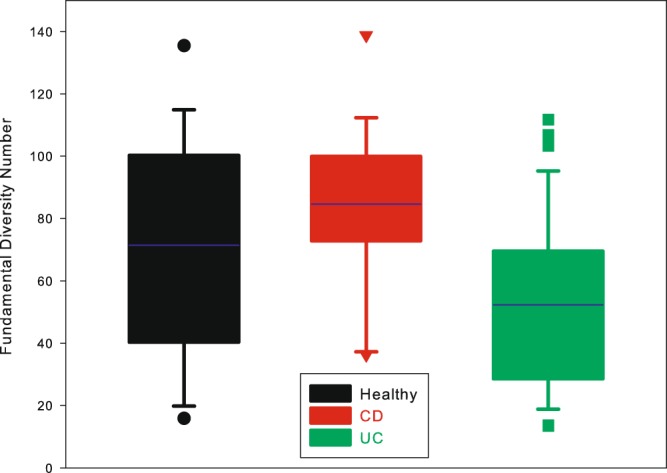
The box plot of the fundamental diversity number (*θ*) in the three treatments (the healthy, CD and UC). The analysis of variance was used to compare the average *θ* value of the three treatments. With the Bonferroni pair-wise comparison, the CD treatment exhibited significant higher *θ* than the UC treatment (*p* < 0.01), but no significant differences were detected in the pairs of CD *vs*. the healthy and UC *vs*. the healthy.

Then we compared the diversity parameters *θ* among 3 treatments. The means of *θ* are 71.43 (95%CI: 54.58–88.28) for the health, 84.59 (95%CI: 72.13–97.05) for CD and 52.29 (95%CI: 43.01–61.57) for UC treatment and the results were showed in Fig. 4. Significant differences were found among 3 treatments *via* variance analysis (*p* < 0.01). In the Bonferroni pair-wise comparison, the mean diversity index for CD was significantly higher than UC treatment (*p* < 0.01) and no significant differences were found between the other pairs.

Several previous studies have reported significant ecological changes of gut microbiota in IBD patients, compared with the healthy individuals. Our study confirmed that IBD is related to the change of gut microbiota diversity as demonstrated by the fundamental diversity number (*θ*) (Fig. 4). We found that the CD treatment showed significant higher biodiversity in gut microbiota that UC treatment. Although CD and UC shared some clinical attributes, they are genetically and fundamentally distinct disease processes^45^. Typically, CD is considered as a systemic disease with a long premorbid phase, where the inflammation could affect any part of the gut, whereas UC is a mucosal disease with an acute onset, often limited to distal colonic tract^45,46^. The compositional difference of gut microbiota between CD and UC patients has been found by previous study^47^. Up to date, though the pathogenesis of IBD has not been fully understood, it has been reported that the loss of protective bacteria and increase in detrimental bacteria occur concomitantly^47^, which may be an important driver for IBD. In the gut microbiota of CD patient, the invasion of harmful bacteria may be the domination factor due to wider scope of inflammation, while in UC patient, the loss of beneficial bacteria may overwhelm the invading bacteria, which may be one explains that CD treatment show significant higher biodiversity that UC treatment.

A series of external factors that influence the gut microbiota are found associated with IBD, for example, larger family size, early life exposure to pets and farm animals, and greater number of siblings are found to increase the risk of IBD, while breastfeeding seems to be protective^9^. These factors may affect, though *via* different ways, the micro-ecosystem of gut, and lead to ecological changes in the gut microbiota. To date, much of the ecological changes associated with IBD have been focused on the composition and diversity of gut microbial communities. The dual objective of our study is to expand existing studies from two aspects. Our first objective is to determine if the spatial heterogeneity of gut microbiota would be changed due to IBD by applying the extended power law models. Our second objective is to investigate whether or not IBD will change the assembly mechanism of gut microbiota by applying the neutral theory.

Regarding our first objective, although previous studies have reported the presence of high inter-individual variations of gut microbiota within humans, the alteration of degree of such variations (*i.e*., spatial heterogeneity) associated with IBD has not been quantified yet. The original Taylor’s (1961) power law^26^ is a powerful tool to measure both the spatial and temporal heterogeneities of population, and the extended power law models (*i.e*., PLEs) by Ma (2015)^27^ are able to assess the heterogeneity at the community level or mixed-specie level. Our study revealed that the Type-I PLE heterogeneity in the healthy treatment is slightly higher than that in the CD and UC treatments. Type-I PLE represents the inter-species heterogeneity, and higher degree of inter-species heterogeneity implies greater differences among species, suggesting higher possibility of the presence of dominant species, which may be a normal state in healthy human gut. We conjecture that the IBD disease (CD or UC) may reduce the abundances of dominant bacterial species and consequently lower the community spatial heterogeneity. Type-III PLE describes mixed-species heterogeneity. It reflects the degree of fluctuations of the abundance of all bacterial species across different samples (*i.e*. intra-species heterogeneity). Because its calculation considers the variance of all species in a set of communities, the changes in several specific species may not cause significant alternation of the intra-species heterogeneity or in another situation, the influence to intra-species heterogeneity caused by the decrease of some species may be compensated by the increase of some other species. The Type-III PLE heterogeneity did not display significant differences among the 3 treatments, suggesting that IBD is not associated with the change of intra-species heterogeneity. The reason may be that IBD could affect only a few certain bacterial species, and such influence is not significant enough to alert the overall intra-species heterogeneity of gut microbiota as there are usually thousands species in gut microbiota. And the loss of beneficial bacteria and acquisition of detrimental bacteria are two opposite processes that may occur parallelly in the gut microbiota of IBD (both UC and CD) patients, from which the influences affecting the intra-species heterogeneity may cancels each other out in some extend.

A number of studies have demonstrated the compositional differences of gut microbiota in IBD patients compared with healthy individuals^48^. As with many ecologists, the gut microbiota is essentially a highly complex community that has little fundamental difference with other ecological communities in nature environment. Hence the ecological theories traditionally developed in the macro-ecology of plants and animals should also be appropriate in micro-ecology area. One of the core topics in community ecology is the mechanism of community assembly and diversity maintenance. However, to the best of our knowledge, the question of whether the ecological force that shapes gut microbiota is related to IBD is still poorly understood. Therefore our second objective in this study is to make efforts to answer this question. We applied Hubbell’s neutral theory for biodiversity to test the neutrality of the observed samples in both IBD patients and healthy individuals. We found that only 4.1% (3/74) community samples satisfied the prediction of the neutral theory, and no significant differences in terms of the passing rate were detected among the three treatments. For this result, we suggest that the assembly and diversity maintenance of gut microbial communities are mostly determined by niche differentiations, and IBD may not significantly influence the assembly mechanism and diversity maintenance of gut microbial communities.

There are two main limitations in our study: (*i*) In previous study, it has been demonstrated that the composition of gut microbiota is different in different parts of gut^28^. Therefore the stool sample may not be the best type for characterizing IBD associated gut microbiota, as it could serve as a pool where the bacteria species come from different parts of gut. Further study should also try to use mucosal sample in the same position across samples to perform parallel comparison. (*ii*) It has been proven that IBD-associated gut microbiota is dynamic^44^. Our study, just like many other studies, used a cross-sectional dataset in a single time point, from which the result may biased due to temporal variance. Further study should make efforts to collect enough time-series datasets and use temporal model to investigate diseases-related ecological changes in gut microbiota.

## Supplementary information

Table S1
